# CO2 lasers in the management of potentially malignant and malignant oral disorders

**DOI:** 10.1186/1758-3284-4-17

**Published:** 2012-04-30

**Authors:** Waseem Jerjes, Zaid Hamdoon, Colin Hopper

**Affiliations:** 1 Department of Surgery, Dijla University College, Baghdad, Iraq; 2 Department of Oral and Maxillofacial Surgery, Al-Yarmouk University College, Baghdad, Iraq; 3 Department of Oral Surgery, Al-Mustansiriya University, Baghdad, Iraq; 4 Department of Surgery, University College London Medical School, London, UK; 5 Leeds Institute of Molecular Medicine, Leeds, United Kingdom; 6 Department of Surgery, Leeds Teaching Hospitals NHS Trust, Leeds, United Kingdom; 7 Oral and Maxillofacial Unit, UCL Eastman Dental Institute, London, United Kingdom; 8 UCLH Head and Neck Centre, London, United Kingdom

## Abstract

The CO_2_ laser was invented in 1963 by Kumar Patel. Since the early 1970s, CO_2_ laser has proved to be an effective method of treatment for patients with several types of oral lesions, including early squamous cell carcinoma.

Laser surgery of oral premalignant disorders is an effective tool in a complete management strategy which includes careful clinical follow-up, patient education to eliminate risk factors, reporting and biopsying of suspicious lesions and any other significant lesions. However, in a number of patients, recurrence and progression to malignancy remains a risk. CO_2_ laser resection has become the preferred treatment for small oral and oropharyngeal carcinomas. Laser resection does not require reconstructive surgery. There is minimal scarring and thus, optimum functional results can be expected.

New and improved applications of laser surgery in the treatment of oral and maxillofacial/head and neck disorders are being explored. As more surgeons become experienced in the use of lasers and as our knowledge of the capabilities and advantages of this tool expands, lasers may play a significant role in the management of different pathologies.

## Introduction

Since the 1970's lasers have been increasingly used in oral and maxillofacial/head and neck surgery. Their effect on tissue is determined by their wavelength and the tissue specific absorption. Lasers can be used for evaporation, excision and coagulation of tissue. The commonly used lasers include carbon dioxide (CO_2_), neodymium:yttrium-aluminium-garnet (Nd:YAG) and Argon lasers. Also, light and laser light are used for the diagnosis of mucosal lesions. For example, by using different excitation wavelength autofluorescence, abnormal lesions can be detected and analysed [1].

Literature in oral and maxillofacial/head and neck oncology continues to support the use of lasers in surgery of premalignant and malignant lesions. Several authorities have explored the indications, techniques, results, benefits and risk issues of lasers used in this field. CO_2_ laser is primarily suited for transoral resections of premalignant and early oral carcinomas. The 5-year survival rates and the postoperative oral function with the carbon dioxide laser are comparable with those obtained following conventional surgical methods. The Nd:YAG laser, because of its unique characteristics (its ability to coagulate and ablate; it is not as precise a cutting tool as the CO2 laser), has specific advantage in the treatment of large oral vascular malformations [2].

The CO_2_ laser was one of the earliest gas lasers invented by Kumar Patel of Bell Labs [3] in 1964, and it still remains the most useful lasers in oral and maxillofacial/head and neck surgery practice. CO_2_ lasers are by far the highest-power continuous wave lasers that are currently available. They are also very useful in oral surgical procedures since the energy is maximally absorbed by water in the oral tissues [1,2]. Laser therapy has been a preferred treatment option for oral leukoplakia since the mid 1980s.

Morphological and functional recovery following laser surgery is superior when compared with conventional cold instrumentation surgery and electrocautery [1,2]. Compared to conventional surgery, epithelial regeneration and wound re-epithelialisation are delayed [1,2] but without any detrimental effect on outcome. Tuncer et al. [4] compared conventional surgery to laser surgery on oral soft tissue pathology. They evaluated the effect of collateral thermal damage on histopathological diagnosis, pain control and postoperative complications. Histological examination of the specimens showed that collateral thermal damage on the incision line did not have adverse effect on the histopathological diagnosis. Pain control was better with lasers. No intra- or postoperative complications were seen in both groups.

Ishi et al. [5] compared the results of partial glossectomy in rabbits: in group one, the excised edges of the wound were left unsutured after partial glossectomy with the CO_2_ laser excision, in group two, the wound was closed after CO_2_ laser surgery. While in group three, the control group, the wound was closed after partial glossectomy with electrocautery. The results were assessed at two, four and eight weeks postoperatively. There was a significant difference in the tongue width between groups 1 and 3 at each time point. There was also a significant difference in the tongue width between groups 2 and 3 at 8 weeks postoperatively. Histologically, the scar tissue of the wound was extensive in the control group, whereas it was localized in the laser group. They concluded that postoperative dysfunction was reduced when excised edges were left unsutured after partial glossectomy with the CO2 laser.

### Advantages of laser management of oral pathology

The use of lasers in the management of oral disorders has been implemented for several years. The advantages of this approach include minimal damage to adjacent tissue, delayed acute inflammatory reaction and reduced myofibroblast activity, leading to reduced wound contraction and scarring. Reduction of collagen in the postoperative phase maintains soft tissue movement. The laser-treated area can be left exposed to granulate, thus obviating any need for skin grafting or wound dressing [1,2].

Since dissection usually follows the approach of ‘en block’ removal of tumour tissue, rather than anatomically based dissection, more normal oral tissues can be preserved. This results in greater preservation of oral function such as swallowing and speech…etc. [1,2,6].

When laser is used, the operating time is reduced. Patients require a shorter hospital stay. The laser procedure is thus cost-effective. Should there be a recurrence or malignant transformation, laser can be used again. Laser usage also does not impose any limitations to implementing multi-modal management with conventional surgery, chemo-radiation and/or photodynamic therapy [1-3,6].

Bornstein et al. [6] assessed 139 patients with 164 intraoral pathological lesions treated with laser. Intra-operative pain control was achieved with topical anaesthesia alone in almost a third of cases. In the remaining 111 lesions, a local anaesthetic was necessary. For pain relief after the operation, 101 patients (72.7 %) used an adhesive wound paste, without any additional oral analgesic. The thermal damage from the laser on the borders of the biopsy specimens did not interfere with the pathologist's establishment of a firm diagnosis. This suggests that laser surgery is an appropriate instrument for excisional biopsies of oral soft tissue lesions.

### Oral premalignant disorders

Hamadah and Thomson [7] assessed the outcome of 78 patients undergoing CO_2_ laser excision of newly diagnosed single oral dysplasias and the influence of clinico-pathological parameters, socio-demographic factors and the presence or absence of residual dysplasia in excision margins. Their results showed that there was no statistically significant association between patients' age, gender, lesion appearance, site of origin, histopathological grading, presence of dysplasia in resection margins, or alcohol consumption and clinical outcome. Smokers, however, were at significantly higher risk of dysplasia recurrence compared to ex-smokers or non-smokers.

Chandu and Smith [8] assessed forty-three patients with73 primary oral leukoplakia for outcome and factors affecting survival. It was postulated that alcohol consumption and previous malignancy are significant prognostic indicators. Continuation to smoking as a risk factor approached significance. Results from this study were comparable to other studies using other conventional modalities.

van der Hem et al. [9] reported on the outcome of a group of 200 patients with 282 oral leukoplakias who were treated by CO_2_ laser evaporation. In the follow-up period (52 months), 251 treated leukoplakias (89.0 %) did not show a recurrence and 28 (9.9 %) local recurrences were observed. In three patients (1.1 %), transformation to squamous cell carcinomas were identified. Although the benefits of CO_2_ laser surgery in the management oral dysplasias have been evaluated, little consideration has been given to the factors which may influence treatment outcome, especially amongst patients developing recurrence or malignant transformation.

Schoelch et al. [10] assessed seventy consecutive laser-treated patients with oral leukoplakia. Thirty-nine patients had some degree of microscopic dysplasia. Six demonstrated high-risk proliferative verrucous leukoplakia. Lasers employed were the CO_2_ and Nd:YAG lasers. The authors reported complete control in 29 patients. 19 patients had small recurrences which were removed with further laser surgery which resulted in control of their disease. 2 patients had total recurrences; and 5 patients developed full-fledged squamous cell carcinoma at the lesion site. Verrucous lesions had an especially high rate of recurrence (83 %), with 9 of 12 ultimately controlled with subsequent surgeries. They concluded that laser is an effective tool for surgery of oral leukoplakia. It plays an important role as a part of an overall management strategy which includes careful clinical follow-up, patient education to eliminate risk factors, report suspicious lesions, and biopsy these and any other significant lesions as appropriate. However, recurrence and progression of untreated areas to cancer remains a risk.

Chiesa et al. [11] retrospectively analysed 167 consecutive patients with oral leukoplakias operated on by CO_2_ laser resection. Within 5 years there were 69 patients with at least one unfavourable event. This included: 31 local relapses, 27 new leukoplakias, 5 oral carcinomas and 6 other neoplasms elsewhere. The Cox regression analysis showed that age of operated patients and size of resected lesions are significantly predictive for development of relapses, new leukoplakias and carcinoma.

### Oral cancer

Surgery continues to be the well established mode of initial definitive treatment for the majority of oral squamous cell carcinoma (OSCC) patients [12]. The aim of surgical ablation for OSCC is the removal of all viable tumour tissue. This intuitively is associated with better overall prognosis. Resection of the primary tumour is employed with dissection and removal of the cervical lymphatic chain, when indicated. Reconstruction of the defect can be by loco-regional repair or by distant free tissue transfer. Radiotherapy plays a key role in the management of early-stage and locally advanced OSCC, either alone or more frequently, combined with surgery and/or chemotherapy [13].

Thirty-seven consecutive patients with cancer of the anterior two-thirds of the tongue without clinical neck lymph nodes or distant metastasis were treated with transoral CO_2_ laser microsurgery. Wang et al. [14] resected the tumour under surgical microscope. Of the 28 patients in the T1/T2 group, 26 patients did not receive postoperative radiotherapy. The local control rate in all 37 patients at 5 years was 93.6 %. No local recurrence occurred in the T1 or T2 cases. Nine patients suffered from neck recurrence and the neck control rate at 5 years was 74.6 %. Eight of these nine patients were salvaged by surgery with adjuvant radiotherapy, and six of them finally achieved disease-free status. The 5-year disease-free survival rate was 88 %.

Ishii et al. [15] compared the rate of recurrence and subsequent metastasis between a group of patients treated with laser surgery and a group treated with radiotherapy (interstitial implant). Laser monotherapy with CO_2_ laser was carried out on 18 cases for squamous cell carcinoma of the mobile tongue. The cure rate of primary tumors was 83.3 %. One patient had subsequent metastasis after laser surgery. No differences were found in the rate of recurrence between the two groups.

Werner et al. [16] reviewed the published data on oncologic laser surgery for the treatment of head and neck carcinomas, along with their own clinical experience with transoral laser surgery for the treatment of carcinomas of the oral cavity, pharynx, and larynx. They concluded that laser surgery has achieved a key position in minimally invasive treatment concepts in the ears, nose, and throat area, and especially for the treatment of malignancies of the upper aerodigestive tract.

Thomson and Wylie [17] reviewed the records of 57 consecutive laser-treated patients presenting over a 4-year period with histologically confirmed dysplastic lesions. Leukoplakias were the commonest clinical lesions (69 %), whilst the floor of the mouth was the most frequent anatomical site (42 %). Laser surgery successfully excised 55 precancerous lesions, 11 of which exhibited more severe dysplasia or neoplasia compared with initial biopsy. Of these patients, 76 % remained disease-free, whilst 24 % developed new dysplastic lesions at distinct or multiple sites, often exhibiting increased dysplasia. Of the patients experiencing recurrence, 7 % developed OSCC, whilst a further 3.5 % presented with other aerodigestive tract cancers.

### Recent studies in our unit

**Figure 1:**
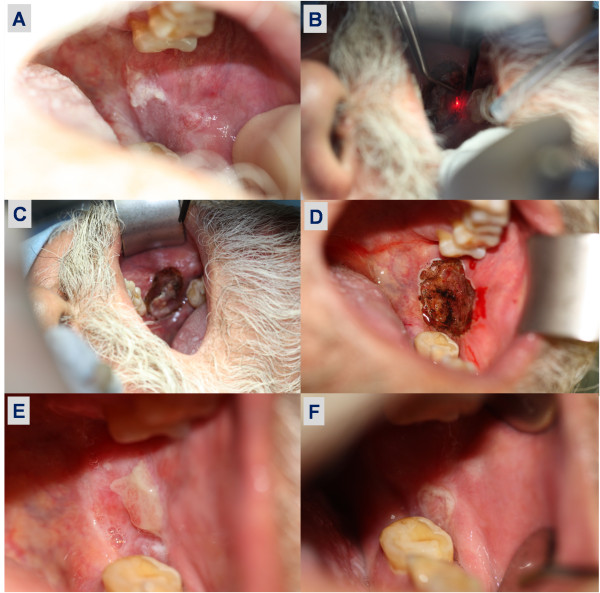
**CO2 laser excision of oral premalignant disorder.** A: patient presented with non-homogenous leukoplakia of the posterior buccal area and the retromolar trigone; B: CO_2_ laser excision of the lesion carried out under general anaesthesia; C: 3 margins (superior, anterior and posterior) have been excised and released prior to release of the inferior margin; D: complete lesion excision and subsequent laser of the base to achieve haemostasis; E: 1 week post excision showing good heeling with minimal frictional keratosis; F: 1 month post excision showing normal oral mucosal with regression of keratosis. *Adapted with permission from: Jerjes W, Upile T, Hamdoon Z, Al-Khawalde M, Morcos M, Mosse CA, Hopper C. CO2 laser of oral dysplasia: clinicopathological features of recurrence and malignant transformation. Lasers Med Sci. 2012 Jan;27(1):169–79.*

In a prospective study carried out at the UCLH Head and Neck Centre [18], a total of 123 oral premalignant disorders from 77 consecutive patients were treated with CO_2_ laser (resection and/or ablation) (Figure 1). The average age was 58 ± 4.8 years. The recovery of all patients was uneventful. Comparisons with the clinical and histopathological features and rate of recurrence as well as malignant transformation were made. The patients were followed-up for a mean of 6.4 years, and biopsies taken where changes suggestive of malignant development were noted.

Homogenous leukoplakias were identified in 31 patients, 34 patients had non-homogenous leukoplakias and 12 patients had erythroplakias. Ex- and lifelong smokers formed 88.3 % of the recruited patients. Current smokers and drinkers formed 55.8 % of the cohort. Erythroplakias were solely identified in heavy lifelong smokers. The most common primary anatomical locations were the lateral border of tongue, floor of mouth and buccal mucosa. Moderate dysplasia was identified in 42 patients while 18 patients showed severe dysplasia.

Laser resection was employed in selected cases (68 patients) and the margins were clear in 53 and showed mild-moderate dysplasia in the involved margins; the rest of the patients had laser ablation. The rate of recurrence had no significant association with the location but the severity of epithelial dysplasia. The rate of first recurrence after laser surgery was 19.5 %. Malignant transformation was observed in 8 patients (10.4 %), in the tongue and the floor of mouth. Recurrence and malignant transformation was mainly identified in erythroplakias and non-homogenous leukoplakias.

**Figure 2:**
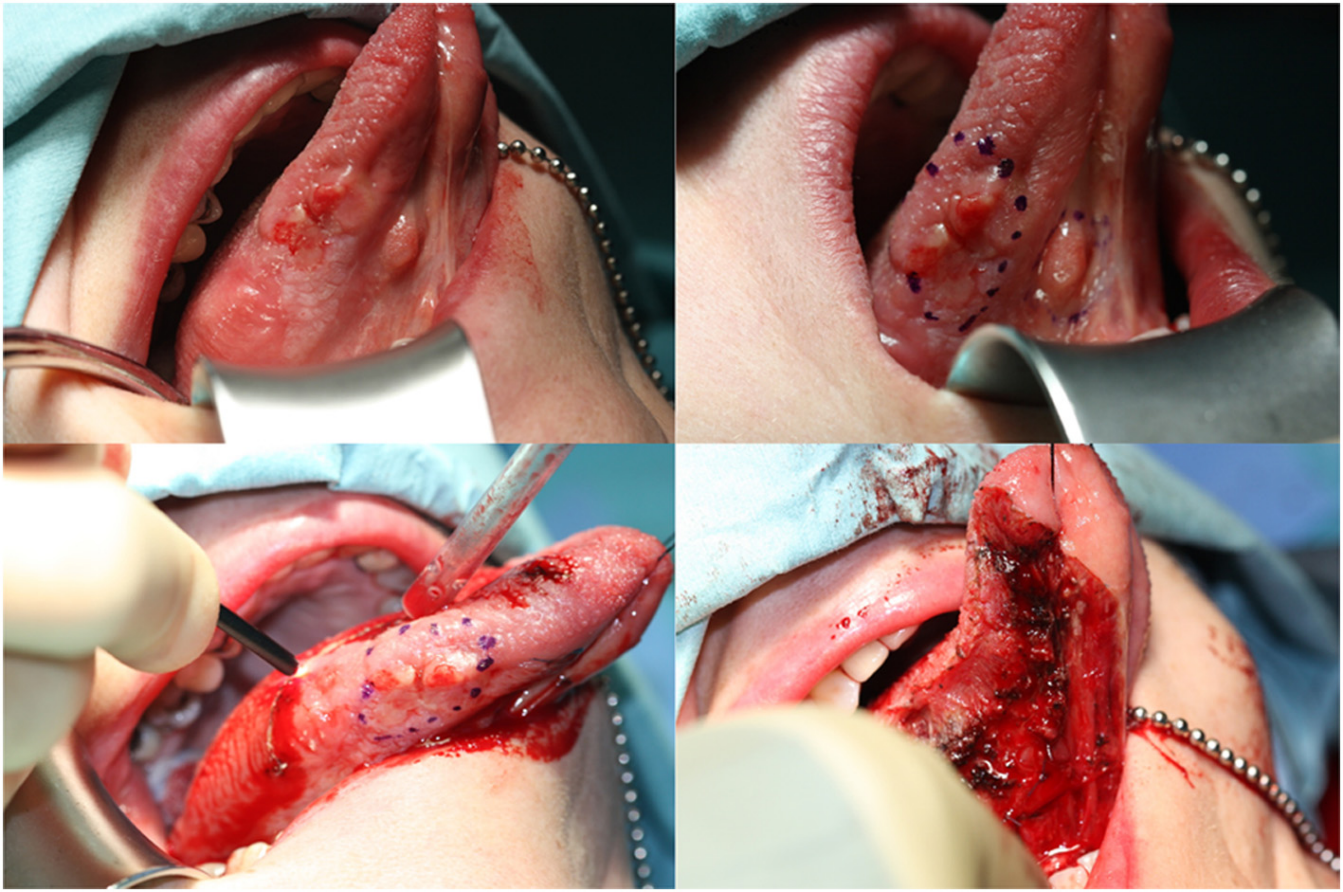
**CO2 laser excision of oral cancer.** Top left: patient presented with a multinodular lesion of the ventrolateral tongue. Incisional biopsy confirmed squamous cell carcinoma. Staging of the patient revealed that there was no nodal involvement or distant metastasis. The patient elected to have the tumor excised with CO2 laser under general anesthesia. Top right: the nodules are identified and marked with an ink before excision. Bottom left: intraoperative CO2 laser excision of the tumor. Bottom right: complete tumor excision and laser of the base to achieve hemostasis. *Adapted with permission from: Jerjes W, Upile T, Hamdoon Z, Mosse CA, Akram S, Hopper C. Prospective evaluation of outcome after transoral CO(2) laser resection of T1/T2 oral squamous cell carcinoma. Oral Surg Oral Med Oral Pathol Oral Radiol Endod. 2011 Aug;112(2):180–7.*

Another prospective study [19] was carried out at the same centre to determine the oncological outcomes following transoral CO_2_ laser resection of T1/T2 N0 OSCC (Figure 2). Three-year disease-specific survival and disease-free survival were evaluated. The data included a range of clinical, operative and histopathological variables related to the status of the surgical margins. Data collection also included recurrence, cause of death, date of death and last clinic review.

Ninety patients (64 males and 26 females) participated in this study. Their mean age at the 1^st^ diagnosis of OSCC was 63.5 years. Two-thirds of the patients were Caucasians. Usually patients presented with ulcers of the tongue, floor of mouth or buccal mucosa. Current and ex-smokers represented 82.2 % of the cohort; while current and ex-drinkers were 78.9 %. Co-morbidities included history of oral lichen planus, oral submucous fibrosis, immunodeficiency, oral dysplasia and/or OSCC. Clinically 81 patients had T1N0 disease while 9 had T2N0 disease.

Pathological analysis revealed that nearly half of the patients had moderately differentiated OSCC, 18 had moderately to poorly differentiated and 19 poorly differentiated carcinoma. Study of the tumour margins showed that the mean depth of invasion was 5.7 mm. Primary tumour clearance was achieved in 73 patients. Recurrence was identified in 11 (12 %) patients. The mean age of 1^st^ diagnosis of the recurrence group was 76.4 years. Most common clinical presentation in the recurrence group was an ulcer involving the tongue or buccal mucosa; most commonly identified in current or ex-smokers or drinkers. Recurrence was identified in patients whom clinical and radiological staging revealed involvement of the locoregional lymphnodes. The surgical margins in this group were also evaluated following re-laser excision or surgical excision ± neck dissection. Follow-up showed a 3-year survival of 86.7 %. Of the twelve patients who died, nine died from non-cancer related causes, two from locoregional spread and one from pulmonary metastasis.

## Discussion

Literature in oral and maxillofacial/head and neck oncology continues to support the use of lasers for surgical removal of malignant lesions of the oral cavity. The 5-year survival rates, and the postoperative function with the laser are at least comparable with those obtained using other surgical methods [2]. The advantages of laser therapy include minimal postoperative pain, conservative site-specific minimally invasive surgeries, and elimination of need for sutures. Laser excision is well tolerated by patients with no intraoperative or postoperative adverse effects. Patients heal with no postoperative loss of function. CO_2_ laser offers equitable surgical option when performing excision of intraoral lesions [20].

Surgical removal of oral premalignant disorders seems one realistic option. Many professionals use scalpel, laser, or cryoprobe as a surgical tool of their choice. The intention of care is to detect, treat and prevent malignant transformation. Several management protocols have been recommended. However, no large trials have shown an ultimate and reliable intervention. No high evidence-based study exists on which to reliably recommend a treatment option. In our opinion, laser excision offers preferred option to electrofulguration for reasons stated above. It has been argued that such operative interventions aggravate dysplasia and that surgical removal of aneuploidic lesions does not improve mortality rates. CO_2_ laser resection has become the elective treatment for small oral and oropharyngeal carcinomas [21].

A further improvement of outcome might be expected by the combination of adjuvant radiotherapy. Pradier et al. [22] reported a study which included 208 patients with advanced squamous cell carcinoma of the head and neck following CO_2_ laser resection, patients were given postoperative radiotherapy. Primary sites included oral cavity, oropharynx, larynx and hypopharynx. In this series, transoral laser surgery in combination with adjuvant radiotherapy in patients with advanced head-and-neck tumours resulted in locoregional control and disease specific survival rates similar to those reported for radical surgery followed by radiotherapy.

The bystander collateral thermal damage of the margin may pose some difficulty in pathological interpretation of laser margins. Photo-coagulation of proteins may mask or alter surface epitopes, rendering some immunohistochemical stain less useful. Obviously a highly experienced sub-speciality head & neck pathologist is an essential member of the team. There is also an uncompromising need for good communication between the operating room and the pathology department. This can range from annotated photo-documentation and narrated video to having physical presence of the pathologist in the theatre room [1,2].

Inadvertent laser damage to the patient or operating theatre staff is an acknowledged risk. However, no data exists to verify the safety margin of commonly employed precautions. A study by Ahmed et al. [23] assessed the safety margins of protective strategies commonly adopted when using lasers to resect tumours of the head and neck. Gauze swabs, neurosurgical patties, surgical gloves, paper drapes and conventional PVC endotracheal (ET) tubes were tested against the following laser variables: power, beam characteristics and angle of beam incidence (90 & 45 degrees). Laser penetration time through the material under test was recorded in seconds (s). The materials were tested dry and some, where appropriate, were tested wet. The results demonstrated dry gauze swabs, neurosurgical patties and paper drapes provided 0 second protection at 2 W (lowest power). However, when wet, the laser failed to penetrate the swabs and neurosurgical patties, even after 180 s of continuous application. Gloves (single or double layer), and ET PVC cuffs were penetrated in less than 1 s at 2 W. Time to penetrate a size 6.0 PVC ET tube at 2 W continuous setting increased from ≪ 1 s at 90 degrees to 42 s at 45 degrees.

In addition to the various oral lesions, the management of patients with sleep apnoea, temporomandibular joint derangements, dental implants and posttraumatic facial scarring has improved significantly with the advent of laser surgery. As further laser systems develop and their technology become more advanced, a thorough understanding of the principles of their use is paramount to ensure safe and effective patient care [24].

## Conclusion

Over the past few decades, the use of lasers among oral and maxillofacial/head and neck surgeons has grown dramatically. Their evolution within the specialty not only has broadened current surgical options for treatment, but also contributed to a variety of new procedures that are now a commonplace in oral and maxillofacial/head and neck surgery.

## Competing interests

The authors declare no competing interests.

## Authors’ contributions

All authors have contributed intellectually and to the writing of this manuscript. All authors read and approved the final manuscript.

## Acknowledgement

We acknowledge the help of TahwinderUpile during the writing up of this article.
